# UV-derived scattering as a high-throughput readout for adeno-associated virus-like particle separation screenings

**DOI:** 10.3389/fbioe.2026.1913731

**Published:** 2026-10-13

**Authors:** Giulia Polazzo, Malte Schwarzer, Jürgen Hubbuch

**Affiliations:** Institute of Process Engineering in Life Sciences, Section IV: Biomolecular Separation Engineering, Karlsruhe Institute of Technology (KIT), Karlsruhe, Germany

**Keywords:** adeno-associated virus (AAV), aqueous two-phase system (ATPS), biomolecular separation, downstream processing (DSP), high-throughput screening, light scattering, UV/Vis absorbance, virus-like particle (VLP)

## Abstract

Virus-like particles (VLPs), including those derived from adeno-associated virus (AAV), are increasingly investigated for vaccination, capsid engineering, and gene-delivery applications. Although precipitation and aqueous two-phase separation (ATPS) represent promising alternatives to adsorption-based capture, their optimization is limited by the lack of rapid analytical methods suitable for complex process samples. Here, VP3-only AAV5 VLPs produced in *Escherichia coli* were used as a model system to evaluate UV-derived light scattering as a high-throughput separation readout. Blank-corrected absorbance at 320 nm was used as a relative descriptor of particulate-associated scattering, while scattering-corrected absorbance at 280 nm monitored the soluble biomolecular fraction. During PEG precipitation, A320 showed condition-dependent agreement with anti-AAV5 ELISA, with the closest correspondence for PEG4000 and increasing divergence at lower PEG molecular weights. Capsid-spiking experiments further showed that dispersed intact capsids alone contributed only marginally to A320, indicating that the signal primarily reflects larger scattering-active structures accompanying capsid depletion. The workflow enabled screening and analysis of 170 precipitation and PEG/phosphate ATPS conditions in biological duplicate within 3 days. Low-molecular-weight PEG systems provided stronger separation between soluble biomolecular material and particulate-associated scattering. In PEG400 ATPS, pH altered the distribution of scattering-active material and capsids between the liquid phases and interphase, whereas selected precipitation conditions showed higher recovery of assembled capsids after resolubilization than ATPS, at the expense of lower impurity removal. Overall, UV-derived scattering provides a rapid, scalable first-line readout for comparing separation conditions and reducing the number of samples requiring capsid-specific and structural characterization.

## 1 Introduction

Virus-like particles (VLPs) are increasingly used in biotechnology, vaccinology, and gene therapy due to their ability to mimic the structural properties of viral capsids while lacking infectious genetic material (Rohovie et al., 2017; Qian et al., 2020). In recent years, adeno-associated viruses (AAVs) and recombinant AAVs (rAAV) have attracted particular interest because of their high stability, low immunogenicity, cell infectivity, and gene expression capability, which increase their relevance in gene delivery and therapeutic vector development (Bennett et al., 2017; Dobrowsky et al., 2021; Fu et al., 2025; Xia et al., 2025). In addition, adeno-associated virus-like particles (AAV-VLPs) are increasingly investigated as model systems for capsid engineering, downstream process optimization, and vaccine development, owing to their lack of viral genome and the possibility to display antigens or peptides on their surface (Le et al., 2019; 2022; Fu et al., 2024).

Established production platforms for rAAV include mammalian cells and insect cells, which are widely used due to their ability to produce functional viral particles (Le et al., 2019; Xia et al., 2025). These systems nevertheless present several limitations, including challenges in large-scale cultivation, the presence of process- and host-cell-derived impurities, and, in the case of insect cell systems, issues related to baculovirus handling and genetic stability. Yeast-based expression systems have also been explored as a potentially more scalable and cost-effective alternative, although their application remains limited by comparatively low production yields (Barajas et al., 2017). More recently, bacterial systems such as *Escherichia coli* *(E.coli)* have been investigated for the production of AAV-VLP, and Le et al. (2022) successfully expressed and assembled VP3-only AAV5-VLPs. These systems offer significant advantages in terms of scalability, cost efficiency, and process simplicity (Le et al., 2019; Fu et al., 2024). While VLPs lack the capability for genome packaging, restricting their direct use for the production of functional AAV vectors, bacterial production nevertheless represents a promising platform and potentially a basis for future strategies involving *in vitro* genome encapsidation (Le et al., 2019). However, the purification of viral particles from complex biological mixtures remains challenging due to the presence of host-cell proteins, nucleic acids, and colloidal impurities with overlapping physicochemical properties (Wang et al., 2019; 2024).

Polyethylene glycol (PEG) precipitation is widely employed as a capture step for viruses and VLPs because of its simplicity, scalability, and compatibility with high-throughput workflows (Ladd Effio and Hubbuch, 2015; Hillebrandt and Hubbuch, 2023; Pons Royo and Jungbauer, 2025). In particular, high-molecular-weight PEG systems (e.g., PEG8000), or high molarity of salts, are commonly used to concentrate viral particles through excluded-volume or salting-out effects (Ladd Effio et al., 2015; Le et al., 2022; Fu et al., 2024; Pons Royo and Jungbauer, 2025). However, these conditions frequently lead to substantial co-precipitation of host-cell impurities or irreversible aggregation of the product, reducing process selectivity and complicating subsequent purification steps such as resolubilization (Ladd Effio et al., 2015; Ladd Effio and Hubbuch, 2015). Aqueous two-phase separation (ATPS) represents an alternative approach in which biomolecules and particles partition between immiscible liquid phases according to their physicochemical properties (Albertsson et al., 1990; Asenjo and Andrews, 2012). In PEG/salt systems, phase behaviour and partitioning are strongly influenced by PEG molecular weight, tie-line lengths (TLLs), pH, and salt composition, all of which affect excluded-volume interactions and phase incompatibility (Asenjo and Andrews, 2012; Silva et al., 2017; Meutelet et al., 2024). These systems were recently successfully applied to rAAVs of different serotypes by Fu et al. (2023) and Fu et al. (2025), showing the potential of low-molecular-weight PEG.

Despite the widespread use of PEG-based systems for viral particle recovery, the systematic optimization of precipitation and partitioning conditions remains limited by the lack of rapid and accessible analytical methods capable of monitoring VLP behaviour in complex mixtures. Conventional approaches typically rely on bulk measurements such as ultraviolet-visible (UV/Vis) absorbance at 280 nm or colorimetric assays, while particle size and aggregation are assessed using turbidity (Dietrich et al., 2024; Wegner and Hubbuch, 2022; Zelger et al., 2016). Complementary methods, including electrophoresis, chromatography, or enzyme-linked immunosorbent assay (ELISA), provide compositional or functional information. However, these techniques are often labor-intensive, low-throughput, or require specialized instrumentation and sample preparation, limiting their applicability for screening large numbers of conditions in complex matrices such as clarified lysates (Zeltins, 2013; Wild, 2013; Lothert et al., 2022). Recent advances, such as Raman spectroscopy-based approaches for monitoring VLP precipitation in crude extracts, highlight the potential of alternative spectroscopic techniques, but often still require specialized instrumentation and data analysis workflows (Dietrich et al., 2024). As a result, comprehensive screening studies involving numerous system compositions become experimentally demanding and difficult to implement routinely.

Light scattering provides an attractive alternative for the indirect monitoring of particulate material in solution. In dispersions containing particles with dimensions comparable to or smaller than the incident wavelength, the measured optical signal comprises contributions from both molecular absorbance and wavelength-dependent scattering phenomena (Bohren and Huffman, 1998). Among the different scattering regimes described in the literature, pure Rayleigh scattering is expected for particles with a characteristic size significantly smaller than the incident wavelength λ , typically below approximately one-tenth of λ (Bohren and Huffman, 1998; Lockwood, 2023). Since AAVs have a characteristic diameter of approximately 25 nm, depending on genome packaging and capsid composition, they lie close to the transition between Rayleigh and Mie regimes, and their scattering contribution can therefore be associated with the presence of dispersed particulate species (Leach and Scheraga, 1960; Basalla and Kendrick, 2023; Xia et al., 2025).

Established light scattering techniques such as dynamic light scattering (DLS) and static light scattering (SLS) are widely used for particle size and concentration analysis in well-defined systems (Tsoka et al., 1999; Makra et al., 2015; Hohl et al., 2017; Pereira Aguilar et al., 2019). However, their application to complex mixtures such as cell lysates is often limited by multiple scattering effects, high background signals, and the presence of heterogeneous particle populations, which compromise data interpretation and reduce throughput (Hohl et al., 2017; Kaur, 2017). In this context, UV/Vis spectroscopy provides a simpler and more robust readout that can be readily applied to complex samples with limited sample preparation or dilution (Mach and Middaugh, 2011; Basalla and Kendrick, 2023). Other major practical advantages include low sample consumption, compatibility with microplate-based laboratory-scale workflows, and straightforward integration into automated high-throughput screening platforms (Sommer et al., 2003; Mach and Middaugh, 2011; Kaur, 2017).

Traditionally, UV/Vis spectroscopy is primarily used to quantify molecular absorbance, while scattering contributions are treated as an interfering background to be corrected, for example, in the determination of protein and nucleic acid concentrations at 280 nm and 260 nm (Basalla and Kendrick, 2023). However, exploiting the scattering contribution as an analytical signal using wavelength regions dominated by scattering to directly monitor particulate behaviour in complex mixtures is not reported. This reveals a key limitation in current downstream processing approaches: the absence of rapid and high-throughput analytical methods that can capture both particulate and soluble behaviour in complex mixtures and support the systematic optimization of separation processes.

In this work, UV-derived scattering was evaluated as a relative descriptor of particulate-associated scattering during PEG-based precipitation and ATPS partitioning, using VP3-only AAV5-VLPs produced recombinantly in *E. coli* as a model system. The relationship between A320 and capsid behaviour was investigated by ELISA, capsid-spiking experiments, and comparison of induced and non-induced lysates. High-throughput screenings were then performed by varying PEG molecular weight, TLL, pH, and salt composition, followed by resolubilization experiments to assess process reversibility. This study evaluates UV-derived scattering as a rapid comparative readout of particulate-associated scattering and soluble biomolecular behaviour across large separation design spaces.

## 2 Materials and methods

### 2.1 Chemicals and stock solutions

All chemicals were purchased from Merck (Darmstadt, Germany), unless otherwise stated. Stock solutions and buffers were prepared using ultrapure water as follows: 70% (w/w) PEG, 40% (w/w) phosphate, and 25% (w/w) sodium chloride solutions. Phosphate stock solutions at pH 6 and pH 8 were prepared by combining 21% (w/w) $NaH_{2}PO_{4}$ with 19% (w/w) $K_{2}HPO_{4}$ , and 5.1% (w/w) $NaH_{2}PO_{4}$ with 34.9% (w/w) $K_{2}HPO_{4}$ , respectively. 80% (w/w) PEG800 was prepared according to the equation reported by Frerix et al. (2006). The lysis buffer consisted of 20 mM Tris, 150 mM NaCl, and 0.001% Pluronic F-127 at pH 8. The resolubilization buffer consisted of 20 mM Tris and 3 M urea at pH 8.

### 2.2 Virus-like particle production in *Escherichia coli*

AAV5-VLPs were produced in *E. coli* BL21 (DE3) cells (New England Biolabs, Ipswich, US-MA) transformed with a pET24b plasmid (Novagen, Merck) encoding only the VP3 capsid protein. The expression construct was cloned by the University of Bielefeld (Cellular and Molecular Biotechnology, Faculty of Technology), which kindly provided the construct described by Le et al. (2022). Protein expression was driven by a T7 promoter system, and transformants were selected using kanamycin. The cultivation, IPTG induction, harvest, and lysis procedures are described in the Supplementary Material. A non-induced control culture was prepared under identical conditions without IPTG induction. Following cultivation, cell pellets from induced and non-induced cultures were equally diluted based on the optical density ${OD}_{600}$ before lysis to ensure comparable biomass input. Pellets obtained from 1 L culture volume were stored at −20 °C. Cell debris was removed after lysis by centrifugation at 4 °C and 17,387 ×g for 40 min, followed by sequential filtration through buffer-prewashed PES syringe filters (0.8 µm, 0.45 µm, and 0.2 µm). The clarified lysate was divided into 5 mL aliquots and stored at −20 °C.

The presence and quantity of VP3-only AAV capsids in the starting material and selected samples during the process were assessed using an AAV5 Titration ELISA kit or the AAV5 Dip’n’Check (both Progen Biotechnik GmbH). According to the assay principle, they recognize a conformational epitope present only in assembled capsids and were therefore used as a capsid-specific, conformation-dependent readout.

### 2.3 Lysate composition using SDS-PAGE

To assess the composition of the clarified lysate, sodium dodecyl sulfate polyacrylamide gel electrophoresis (SDS-PAGE) was performed using NuPAGE™ 4%–12% Bis-Tris protein gels with LDS sample buffer, MES running buffer, Mark12™ protein standard (all Thermo Fisher Scientific Inc.), and 50 mM dithiothreitol (DTT) in the sample solution. Electrophoresis was carried out using a PowerEase500 power supply (Thermo Fisher Scientific) for 30 min at 200 V. Proteins were subsequently stained using Coomassie blue staining solution (InstantBlue®, Abcam).

### 2.4 High-throughput precipitation and partitioning experiments on a liquid handling station

A Tecan Freedom EVO system (Tecan, Crailsheim, Germany) was used as a liquid handling platform for automated precipitation and ATPS experiments. The system setup and calibration procedure have been described previously (Oelmeier et al., 2011). The robotic workstation was controlled using EVOware Standard 2.5. Pipetting volumes were imported and photometric data were exported using Excel 2007 (Microsoft Corporation, Redmond, WA), while MATLAB R2016b (MathWorks) was used for tie-line determination.

#### 2.4.1 Precipitation

An automated procedure previously developed in our group was used for the precipitation screening (Oelmeier et al., 2013; Ladd Effio and Hubbuch, 2015), with minor modification. Phosphate pH 8 or PEG were added individually to screen the solubility of the particles and host-cell impurities in single-phase systems, where precipitation was induced by phosphate or PEG only, with 2% (w/w) NaCl as an additive. See Table 1 for a summary of the conditions screened. The precipitant solubility was screened between 0% and 24% (w/w) in steps of 2% or 3%. A total of 250 µL was prepared in a 96-well protein low-bind plate (Greiner Bio-One GmbH, Kremsmünster, Austria), following the same procedure as for the ATPS screening, using 57.7 µL clarified lysate to maintain a constant lysate-to-total-volume ratio throughout the study and extending the centrifugation step by 15 min. A total of 150 µL of supernatant was collected for analysis. For the resolubilization test, 250 µL of resolubilization buffer was added to each well after complete removal of the supernatant. Precipitation experiments were evaluated using spectral analysis for the liquid phase. Apparent solid-associated fractions were estimated from the normalized signal not recovered in the liquid phase relative to the clarified-lysate reference.

**TABLE 1:** Summary of partitioning conditions investigated, varying system components, PEG molecular weight, NaCl concentration, pH, and tie-line length (TLL) or precipitant concentration.

Partitioning method	System components		NaCl % (w/w)	Conditions % (w/w)
Aqueous two-phase systems	PEG400 PEG600 PEG800 PEG1000	Phosphate pH 8 Phosphate pH 6	0, 2	TLL 20, 25 30, 35 40, 45 50, 55
Precipitation	PEG400 PEG600 PEG800 PEG1000 Phosphate pH 8		2	Precipitant 0–25

A preliminary precipitation screening with PEG4000 (0%–12% (w/w)) and 2.5% NaCl was used as a proof-of-concept system to validate the analytical workflow. This condition was not included in the systematic screening presented in this study, but was selected as a representative case. The validation was then extended to PEG400 (0%–25% (w/w)), PEG1000 (0%–20% (w/w)), and PEG4000 (0%–15% (w/w)) with 2% (w/w) NaCl as an additive.

#### 2.4.2 ATPS partitioning

An automated titrimetric method for binodal curve and tie-line determination was developed by our group and adapted (Oelmeier et al., 2011; Bensch et al., 2007). The cloud point method was used to identify the binodal curves, which were fitted to the Merchuk equation (Merchuk et al., 1998) (Equation 1):

$$c_{\mathrm{PEG}}=A\cdot exp\left(B\cdot c_{\mathrm{salt}}^{0.5}+C\cdot c_{\mathrm{salt}}^{3}\right).$$ (1)

Tie-lines were determined using a colorimetric method with water, phosphate, PEG, additive, and methyl orange as the colorant, as developed by Diederich et al. (2013) and adapted by Meutelet et al. (2024), with modifications. See Supplementary Material for the detailed procedure.

A total of 128 different ATPS conditions using PEG/phosphate systems were investigated by varying PEG molecular weight (400, 600, 800, 1,000), TLL (20–55), pH (6–8), and NaCl concentration (0%-2% (w/w)) as an additive, in order to evaluate the distribution of particulate-associated scattering and soluble biomolecular signals across the ATPS phases, complemented by capsid-specific analysis of selected samples. A total of 650 µL was prepared in a 1 mL 96-deep-well Protein LoBind plate (Eppendorf SE, Hamburg, Germany) by adding the components in the following order: water, phosphate, PEG, additive, and 150 µL clarified lysate. Phosphate and NaCl stock solutions were filtered through a 0.2 µm PES filter prior to use. After vigorous manual shaking to enhance vertical mixing in the deep-well plate, the plate was shaken at 1,100 rpm for 15 min on an orbital shaker and then centrifuged at 2,916 ×g for 15 min to achieve phase separation. Aliquots of 200 µL from both the top and bottom phases were collected for analysis using a pipette. To sample the bottom phase, the dense interphase was gently displaced along the well wall, allowing retrieval of the bottom phase from the lowest part of the well. To isolate the interphase, all residual liquid phases were removed with a pipette, as the interphase remained attached to the wall. For the resolubilization test, 650 µL of resolubilization buffer was added after removal of the liquid phases. The solution was then thoroughly mixed by pipetting, shaken on an orbital shaker for 60 min at 300 rpm, and centrifuged for 15 min at 4,000 × g to remove the insoluble fraction. Subsequently, 200 µL of the supernatant was collected for analysis. A summary of all screened conditions is provided in Table 1, corresponding to a total of 128 ATPS conditions tested in biological duplicate.

### 2.5 UV/Vis acquisition

A UV/Vis spectrometer (Spark, Tecan Group Ltd.) was used to record absorbance spectra in the range 200–500 nm, with a 2 nm step size, in order to capture both molecular absorbance and light scattering contributions of the samples. A volume of 40 µL of sample was transferred to a SpecPlate (PHABIOC GmbH, Karlsruhe, Germany), which enables the simultaneous measurement at four optical path lengths (0.1 mm, 0.7 mm, 1.4 mm, 2.0 mm) while simultaneously minimizing artefacts related to meniscus formation (Radtke, 2026). For each system point, performed in biological duplicate, two replicates were measured together with one blank consisting of the same concentrations of system components without lysate. Blank spectra were used for background subtraction.

#### 2.5.1 Scattering correction

The measured signal at a wavelength λ was assumed to consist of a chromophoric absorption term and a scattering contribution arising from suspended particles and aggregates, which introduces an additional wavelength-dependent contribution to the apparent absorbance and can bias Beer–Lambert-based interpretation (Pace et al., 1995; Grimsley and Pace, 2003; Thakkar et al., 2012; Basalla and Kendrick, 2023). With the objective of using this method on clarified lysate, absolute concentration determination based on a single Beer–Lambert absorptivity is not appropriate (Kaur, 2017). We therefore use the scattering-corrected absorbance at 260 nm (A260) and 280 nm (A280) as relative indicators of nucleic acids and proteins. In this context, the absorbance at 280 nm cannot be interpreted as a purely protein-specific signal due to the high nucleic acid content of the clarified lysate. Instead, it was considered as a composite indicator of the soluble biomolecular fraction, including both proteins and nucleic acids. The corrected absorbance values A260 and A280 were also used to compute the A260/A280 ratio, which served as an indicator of the relative contribution of host-cell nucleic acids with respect to the soluble biomolecular fraction. The A260/A280 ratio was used only as a relative compositional indicator and not as a quantitative measure of DNA concentration. In the complex clarified lysate, it cannot distinguish free DNA from RNA, protein-associated nucleic acids, or other nucleic-acid-containing species.

Since AAV-VLPs have a characteristic diameter of approximately 25 nm, the generalized power-law model $A_{\mathrm{scatt}}\left(\lambda \right)=k\lambda^{\left(-n\right)}$ was adopted to account for a possible mixed contribution of Rayleigh and Mie scattering (Leach and Scheraga, 1960; Basalla and Kendrick, 2023). The scattering intensity k and the scattering exponent n were estimated by fitting the spectra in the 310–340 nm region, where molecular absorbance contributions are negligible, using a log–log linearization approach (Alyones et al., 2019; Basalla and Kendrick, 2023). In PEG-containing systems, high polymer concentrations can affect the measured UV signal through changes in the optical background, including increased light scattering and refractive index effects (Asenjo and Andrews, 2012). To account for these contributions, the scattering component was independently estimated for both sample and blank by fitting their raw spectra. The blank-subtracted absorbance $A_{\mathrm{net}}$ was then further corrected by removing the differential scattering contribution between sample and blank $\Delta A_{\mathrm{scatt}}$ , yielding the corrected absorbance (Equation 2):

$$A_{\mathrm{corr}}\left(\lambda \right)=A_{\mathrm{net}}\left(\lambda \right)-\Delta A_{\mathrm{scatt}}\left(\lambda \right).$$ (2)

The same optical path length of 0.1 mm was used consistently for both scattering fitting and absorbance evaluation to ensure internal consistency of the correction procedure. For comparative analysis across different conditions, the A280 signal of liquid phases was further normalized with respect to a reference clarified lysate. The solid phase values were calculated by mass balance with respect to the reference lysate. All spectral processing, including scattering fitting, absorbance correction, and normalization, was performed using a custom Python script, ensuring a consistent and automated analysis workflow across all samples.

#### 2.5.2 A320 as a descriptor of particulate-associated scattering

For solutions where the scattering exponent n approaches the Rayleigh regime (n∼4) , the signal above 300 nm is dominated by light scattering and can be associated with dispersed particles and aggregates in solution (Leach and Scheraga, 1960; Bohren and Huffman, 1998; Basalla and Kendrick, 2023). While spectral fitting was required to accurately estimate and subtract the scattering contribution across the full wavelength range, a single-wavelength readout was preferred as a robust descriptor for comparative analysis of complex mixtures. Therefore, the blank-corrected absorbance at 320 nm (A320) was used as a proxy for particulate-associated scattering in liquid phases. This wavelength minimizes residual molecular absorbance while maintaining a sufficiently high signal-to-noise ratio. For this purpose, the signal obtained at a path length of 2.0 mm was used to further improve sensitivity. As this path length differs from that used for scattering fitting (0.1 mm), the A320 signal was normalized to a reference lysate. Min-max normalization was applied to scale the A320 signal between 0% and 100%, using the clarified lysate as the upper scattering reference and a PEG800 condition with no detectable capsids by Dip’n’Check (detection range 1.0E+09 to 1.0E+11 capsids/mL) as an empirical lower scattering reference. This procedure expresses A320 as a relative measure of particulate-associated scattering and facilitates comparison between conditions, but does not define an absolute zero-scattering or particle-free state.

It should be noted that the measured A320 signal represents the combined optical contribution of all particulate species present in the sample. As light scattering depends on both particle concentration and particle size (as well as particle size distribution), the measured signal reflects the overall particulate scattering of the system rather than these individual parameters separately. Consequently, A320 cannot distinguish between individual VLPs, soluble aggregates, or other particulate species, but provides a comparative descriptor of the combined scattering signal generated by these particulate species under identical measurement conditions. Analogous to the A280-based analysis, the apparent missing normalized A320 signal relative to the reference lysate was calculated to estimate the redistribution of particulate-associated scattering between the liquid and solid phases. This derived quantity reflects relative scattering intensity only and should not be interpreted as a quantitative particle mass balance or particle recovery.

To further assess the contribution of intact capsids to the UV-derived scattering signal, a spiking experiment was performed using a clarified non-induced lysate as background matrix. Purified AAV5 empty capsids (AAV5 empty capsids reference material, Progen Biotechnik GmbH) were serially diluted to concentrations ranging from $4.4\times 10^{11}$ to $4.4\times 10^{9}$ capsids ${\text{mL}}^{-1}$ and added to a fixed amount of clarified lysate. UV/Vis spectra were acquired under the same conditions as described above, and the absorbance at 280 nm and 320 nm was evaluated to investigate the influence of intact capsids on the biomolecular absorbance and particulate-associated scattering signals, respectively. This experiment was designed to evaluate the contribution of intact capsids to the overall scattering signal in a complex lysate background independently of changes in host-cell-derived particulate material.

## 3 Results

### 3.1 Lysate composition

To assess the composition of the clarified lysate, SDS-PAGE was performed. The clarified material, diluted 1:40, was loaded alongside a protein marker (see Supplementary Material, Supplementary Figure S1). The large number of bands indicates that the clarified lysate contains a wide range of protein species.

### 3.2 Preliminary experiments for UV-derived scattering

To evaluate the relationship between UV-derived scattering and capsid recovery during precipitation, the A320 signal was compared with independent ELISA-based capsid quantification. A total capsid titer of $4.19\times 10^{11}$ cp/mL with a relative standard deviation of 5.7% was found in the clarified starting material. For the representative PEG4000 condition, normalized ELISA recovery and A320 exhibited highly similar concentration-dependent depletion profiles (Figure 1A). A strong correlation was observed between the two measurements (Pearson r=0.995 , $R^{2}=0.990$ , n=8 , $p<10^{-6}$ ; Figure 1B).

**FIGURE 1:**
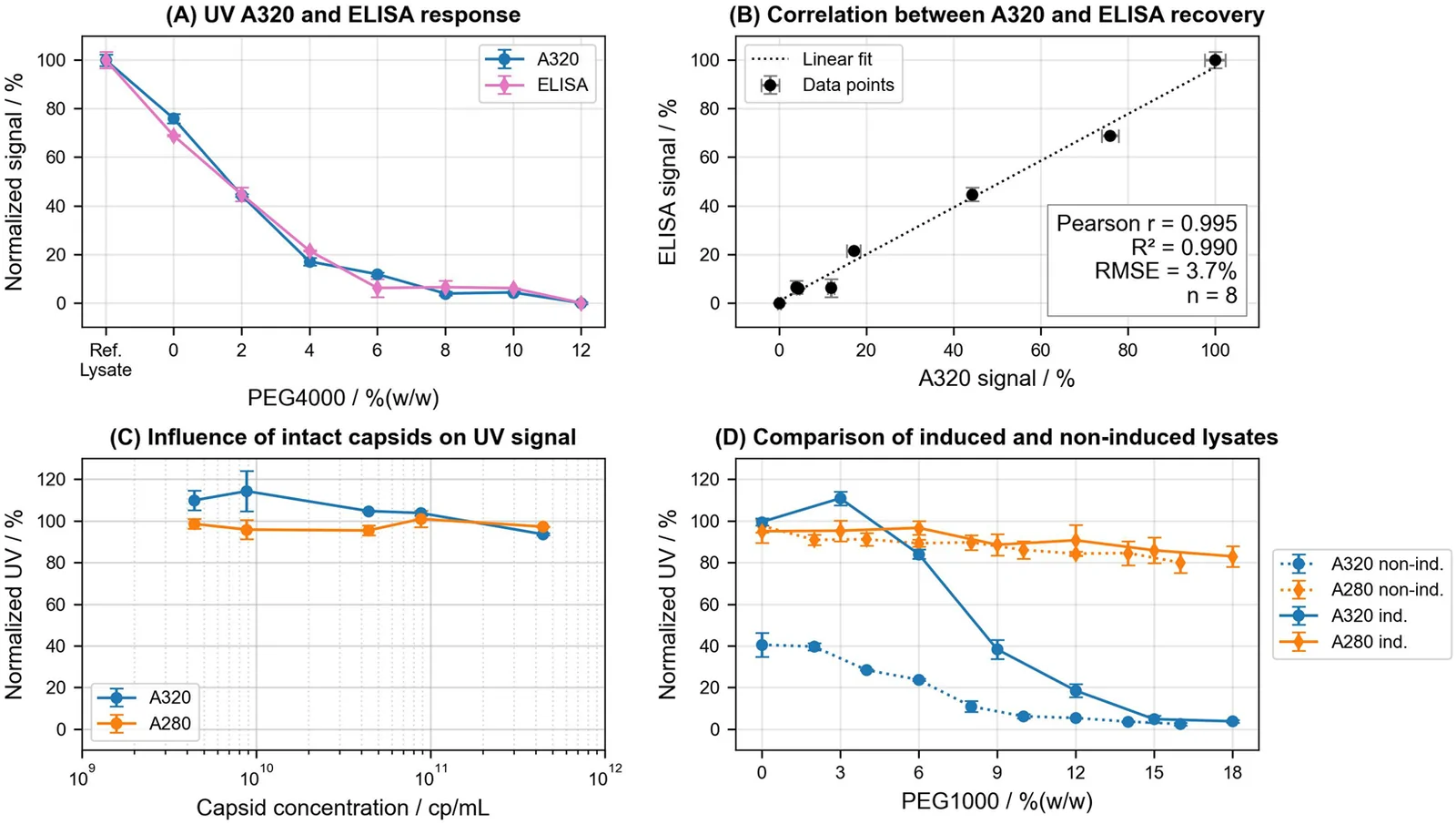
Comparison of UV-derived particulate-associated scattering (A320) with anti-AAV5 ELISA during PEG4000 precipitation **(A,B)**, effect of purified AAV5 empty-capsid spiking on UV signals in non-induced clarified lysate **(C)**, and comparison of induced and non-induced lysates during PEG1000 precipitation **(D)**. **(A)** A320 and ELISA exhibit similar concentration-dependent depletion profiles under the investigated PEG4000 condition. **(B)** Linear association between normalized A320 and ELISA signals. **(C)** No measurable changes in A280 or A320 were observed across the investigated capsid concentrations. **(D)** Induced and non-induced lysates show similar concentration-dependent trends, while the induced lysate exhibits consistently higher A320 values. Data are normalized to the corresponding clarified lysate before precipitation.

The contribution of intact capsids to the UV-derived scattering signal was assessed by spiking purified AAV5 empty capsids into a clarified non-induced lysate over a concentration range of $4.4\times 10^{11}$ to $4.4\times 10^{9}$ capsids ${\text{mL}}^{-1}$ (Figure 1C). Neither A280 nor A320 showed measurable changes across the investigated capsid concentrations. While the constant A280 was expected due to the unchanged lysate composition, the absence of detectable changes in A320 indicates that dispersed intact capsids alone do not substantially contribute to the UV-derived scattering signal under the investigated conditions. Taken together, these results show that, although A320 tracked capsid depletion under the investigated PEG4000 condition, it does not directly represent capsid concentration.

Finally, a PEG1000 2% NaCl screening was performed using both induced and non-induced clarified lysates, to assess the precipitation behaviour of the signals with or without VLPs. Both samples exhibited similar decreasing trends in A280 and A320 with increasing PEG concentration (Figure 1D). While the overall profiles were comparable, the induced lysate consistently exhibited higher A320 values than the non-induced lysate throughout the investigated PEG concentrations, indicating a higher particulate-associated scattering signal under induced conditions.

### 3.3 Precipitation screening

A precipitation screening was performed to investigate the behaviour of the soluble biomolecular fraction (A280) and the particulate-associated scattering signal (A320) in single-phase systems. Different precipitant agents (phosphate or PEG) and PEG molecular weights (PEG400-PEG1000) were screened by increasing the precipitant concentration (Table 1), and the results were analysed in terms of normalized UV signals (Figure 2). In Figure 2A, the scattering signal A320 exhibits a sharp decrease already at low precipitant concentrations, indicating rapid depletion of the scattering-active material from the supernatant. At higher precipitant concentrations, the A320 signal reaches an empirical lower plateau, with no further measurable decrease upon increasing precipitant concentration. In contrast, the A280 signal remains relatively constant over the same concentration range and decreases only gradually at higher precipitant concentrations. The A260/A280 absorbance ratio in the range of 1.9–2.0 across all conditions, consistent with the value measured for the clarified lysate, further suggests that their relative UV contributions remain approximately constant during precipitation (see Supplementary Material for all investigated systems).

**FIGURE 2:**
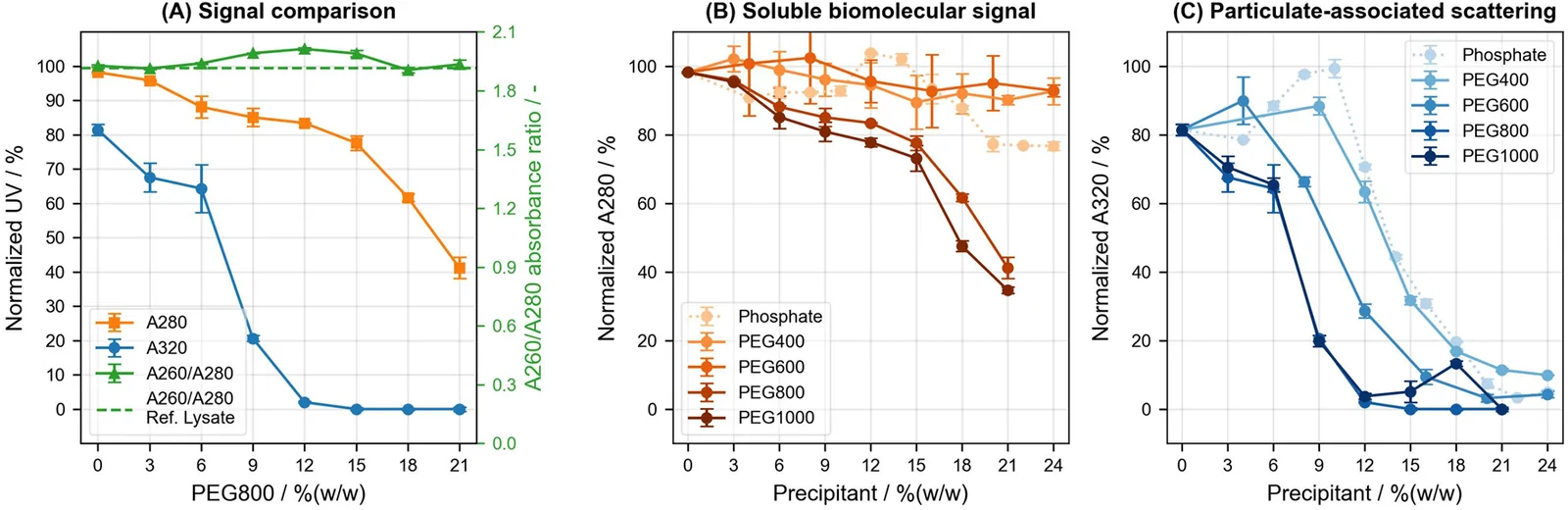
Precipitation behaviour of the soluble biomolecular signal (A280), A260/A280 absorbance ratio, and particulate-associated scattering signal (A320) as a function of precipitant concentration. **(A)** The representative profile for PEG800 shows a faster decrease of the scattering signal (A320) compared to biomolecular absorbance (A280), while the A260/A280 absorbance ratio remains constant. **(B)** The A280 signals for different precipitants indicate a gradual reduction of the soluble biomolecular signal with increasing precipitant concentration. **(C)** The A320 signals show a steeper decrease with increasing precipitant concentration and a clear dependence on PEG molecular weight. Data are normalized to the corresponding clarified lysate before precipitation.

A comparison between different precipitants reveals a clear effect of PEG molecular weight on precipitation behaviour (Figures 2B,C). Low-molecular-weight PEG400-PEG600 and phosphate show a weaker impact on both A280 and A320 signals compared to higher PEG molecular weights. Specifically, the A280 signal decreases gradually with increasing precipitant concentration, reaching approximately 40% of the initial value for PEG1000, while higher residual A280 values are observed for PEG400, PEG600 and phosphate across the investigated range (Figure 2B). In contrast, the A320 signal decreases more steeply with increasing precipitant concentration and reaches the empirical lower plateau at high PEG concentrations, with higher molecular weights inducing an earlier and more pronounced decrease of the particulate-associated scattering signal from the supernatant (Figure 2C). For phosphate precipitation, an initial increase in A320 is observed in the low concentration range (0%–10%), indicating a transient increase in particulate-associated scattering prior to its subsequent reduction.

Selected samples from the late slope and plateau region of the A320 curve were analysed using Dip’n’Check. Capsids were detected in the range of 15%–20% PEG400 and 6%–12% PEG800, whereas no capsids were detected at 15% PEG800, indicating complete depletion of detectable capsids from the supernatant. This condition was therefore selected as the empirical lower reference for min–max normalization of the scattering signal across all partitioning systems. The resulting 0% reference represents the scattering level of this selected condition and should not be interpreted as an absolute zero-scattering state, a particle-free condition, or an analytical detection limit. For phosphate-induced precipitation, Dip’n’Check analysis was performed in the range of 12%–20%, consistently resulting in no detectable capsids. The continued evolution of A320 despite the absence of detectable capsids therefore cannot be assigned specifically to VLP behaviour. It may reflect either an underestimation of capsids caused by the phosphate-rich assay matrix or the continued aggregation and depletion of non-VLP scattering-active material after detectable capsids have already been removed from the supernatant. Despite this, the A320 signal exhibited a trend similar to that observed for PEG-induced precipitation, and the same min-max normalization approach was therefore applied to phosphate systems and interpreted as a relative rather than absolute scaling procedure.

To further evaluate the relationship between UV-derived scattering and capsid recovery, selected supernatant samples from PEG400, PEG1000, and PEG4000 precipitation screenings were additionally quantified by ELISA (Figure 3). For all three PEG molecular weights, A320 and ELISA showed the same overall tendency toward earlier depletion with increasing PEG molecular weight. The correspondence was closest for PEG4000 and remained good for PEG1000, whereas PEG400 showed a distinct concentration-dependent divergence between the two readouts. At low PEG400 concentrations, A320 initially remained constant or increased while ELISA-detectable capsid recovery decreased. At higher concentrations, A320 approached its empirical lower plateau while a substantial capsid fraction remained detectable in the supernatant. These results show a progressive decoupling between particulate-associated scattering and capsid precipitation at low PEG molecular weight. Based on their distinct UV-derived separation profiles, selected conditions of 21% PEG400 with 2% NaCl for purity and 15% PEG1000 with 2% NaCl for partitioning efficiency were chosen to assess impurity and reversibility in solid phase resolubilization experiments, as summarized in Table 2.

**FIGURE 3:**
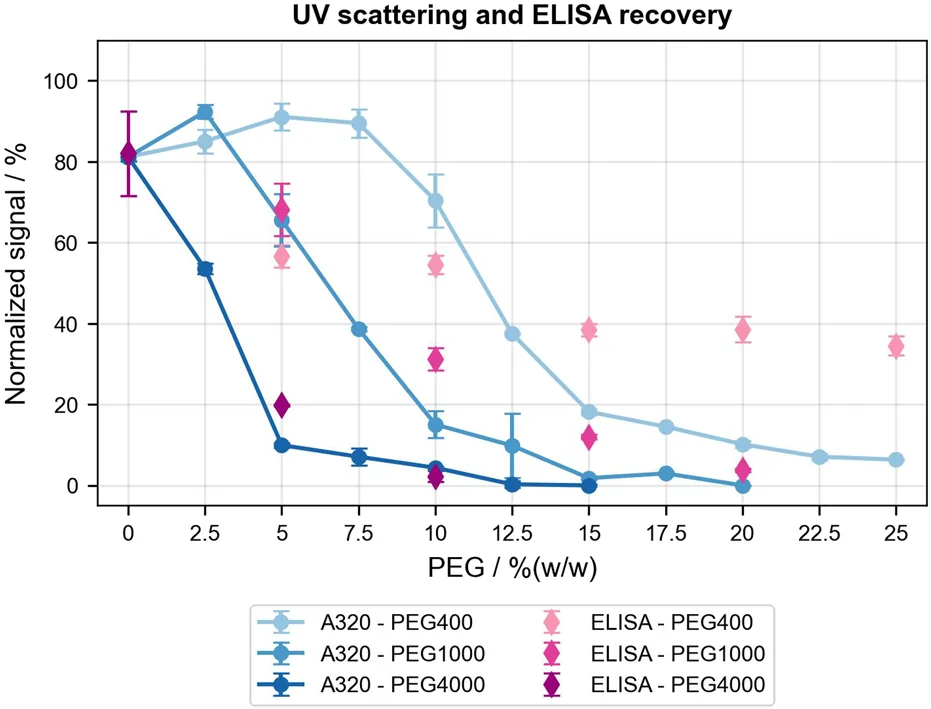
Comparison of capsid recovery measured by anti-AAV5 ELISA and particulate-associated scattering (A320) during PEG precipitation. Both measurements show progressively earlier depletion with increasing PEG molecular weight, while their divergence increases at lower PEG molecular weight. Data are normalized to the corresponding clarified lysate before precipitation.

**TABLE 2:** Performances of selected precipitation and aqueous two-phase separation (ATPS) conditions in terms of partitioning and resolubilization.

Quality attributes	Precipitation		ATPS	
​	21% PEG400 2% NaCl	15% PEG1000 2% NaCl	16.6% PEG400 25.4% phosphate pH 6	20.6% PEG400 18.9% phosphate pH 8
Partitioning in liquid phases				
Capsid recovery	39%	12%	6%	15%
Residual A320 signal	13%	5%	9%	11%
A280 signal retained	89%	81%	92%	97%
Resolubilization				
A280-based soluble fraction	100%	53%	40%	76%
A260/A280 absorbance ratio	1.50	1.68	1.26	1.21
Capsid recovery	49%	47%	21%	11%

The values refer to normalized UV signals at 280 nm (soluble biomolecular fraction) and 320 nm (particulate-associated scattering), and anti-AAV5 ELISA recoveries compared to the clarified lysate. Bottom-phase samples were below the ELISA detection limit and are not included in the reported ATPS recovery.

### 3.4 ATPS screening

Phase diagrams for sixteen different PEG/salt systems were generated using automated routines on a liquid handling station (Supplementary Figure S2). Binodal coefficients, system points obtained, and comments on the results are reported in the Supplementary Material. Based on the identified phase diagrams and system compositions, ATPS screenings were conducted to evaluate the distribution of the biomolecular fraction (A280) and scattering-active material (A320) across the different phases. Representative results are shown in Figure 4, while a summary of all the partitioning behavior of the systems can be found in Supplementary Figure S3. TLLs and system points corresponding to single-phase conditions are not shown or appear as empty entries.

**FIGURE 4:**
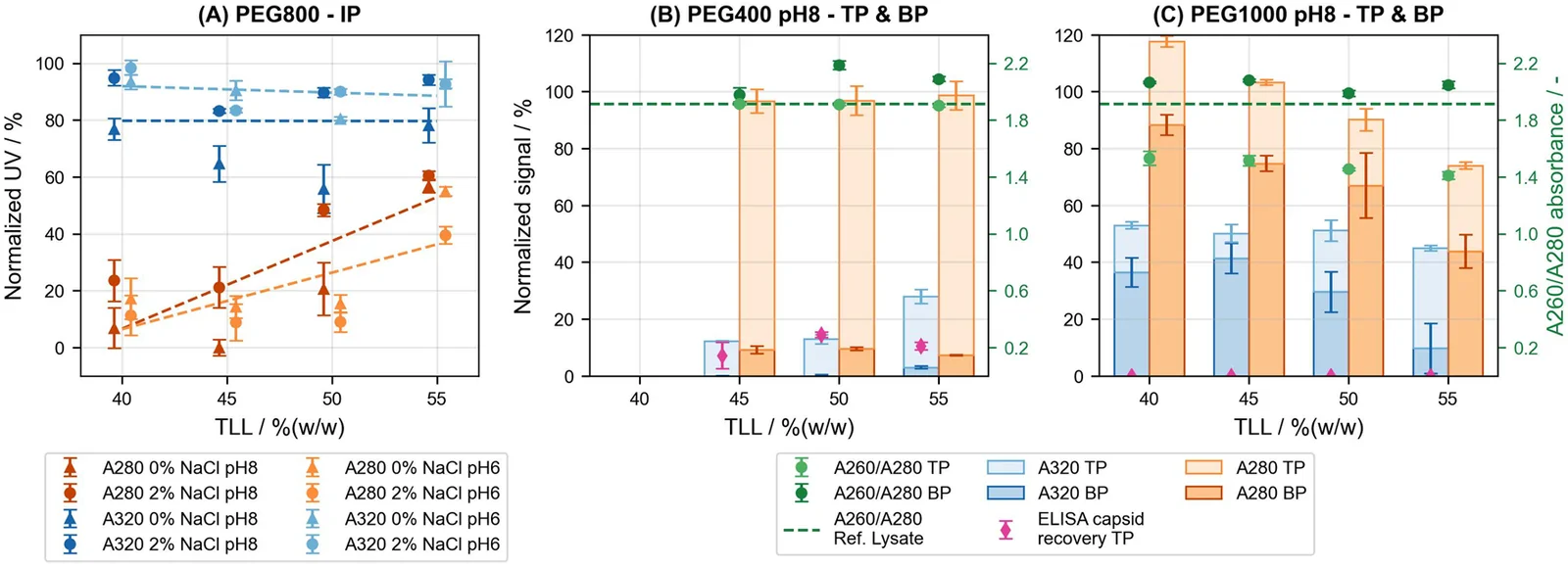
UV-derived partitioning profiles and selected capsid recoveries in PEG/phosphate ATPS. Apparent interphase-associated A280 and A320 values were calculated from the missing normalized liquid-phase signal relative to the reference lysate. **(A)** The distribution of UV signals in the interphase (IP) for PEG800 systems shows that increasing tie-line length (TLL) reduces separation between soluble biomolecular fraction (A280) and particulate-associated signal (A320), while NaCl promotes increased interphase-associated A280. Systems at pH 6 show higher apparent interphase-associated A320 compared to pH 8. **(B,C)** Distribution of UV signals in top phase (TP) and bottom phase (BP) for PEG400 and PEG1000 at pH 8 indicates that low PEG molecular weight enables stronger separation between the soluble biomolecular fraction and scattering-active material, whereas higher molecular weight promotes redistribution toward the bottom phase. Anti-AAV5 ELISA detected capsids in PEG400 top-phase samples, whereas PEG1000 top-phase samples and all analysed bottom-phase samples were below the ELISA detection limit. The A260/A280 absorbance ratio is consistent with a greater relative nucleic-acid-associated contribution in the bottom phase.

The distribution of normalized A280 and A320 signals in the interphase for PEG800 systems is presented in Figure 4A, where scatter points represent the single systems and the dashed lines are the overall trends of systems with the same pH. A dependence on TLL and salt composition is observed. The addition of NaCl leads to an increased fraction of both host-cell-derived biomolecular material and particulate-associated material in the interphase, consistent with enhanced salting-out effects. Increasing TLL results in a progressive increase of A280 in the interphase, while the A320 signal remains comparatively stable. Systems at pH 6 show similar overall trends compared to pH 8, but with an increased fraction of scattering-active material in the interphase.

From the perspective of the liquid phases, the normalized A280 and A320 signals in the top and bottom phases are shown as stacked clustered barplots in Figures 4B,C for systems at pH 8 without NaCl. A clear redistribution of both signals is observed as a function of PEG molecular weight. At PEG400, a strong separation is observed, with the soluble biomolecular fraction predominantly remaining in the top phase and the A320 signal being almost completely depleted. In contrast, increasing the PEG molecular weight leads to a shift of both A280 and A320 signals from top phase and interphase toward the bottom phase. Selected top and bottom phase samples were also analysed by ELISA. Measurable capsid recoveries were observed in PEG400, PEG600, and PEG800 top-phase samples, with a progressive decrease in detectable recovery with increasing PEG molecular weight and TLL. For PEG1000, most top-phase samples were below the ELISA detection limit under the tested assay conditions, together with all the analysed bottom-phase samples. The A260/A280 ratio in the top phase decreases with increasing PEG molecular weight and TLL, while the ratio in the bottom phase remains approximately constant around 2 across all conditions. This is consistent with a reduced relative nucleic-acid-associated contribution in the top phase, while the relative composition of the bottom phase remains stable. Based on the UV-derived partitioning profiles, PEG400 at TLL50 and pH 6 or 8, together with PEG1000 at TLL50 and pH 8, were selected as representative ATPS conditions for resolubilization and capsid-specific analysis.

### 3.5 Resolubilization of solid phases and capsid-specific recovery

Selected precipitation and ATPS conditions were further evaluated to compare resolubilization of the biomolecular fraction with recovery of ELISA-detectable assembled capsids (Figure 5; Table 2). A 3 M urea buffer was used to resolubilize the isolated pellets or interphases, as a preliminary screening showed that a resolubilization buffer with a lower amount of urea was not sufficient to resolubilize the pellet (data not shown).

**FIGURE 5:**
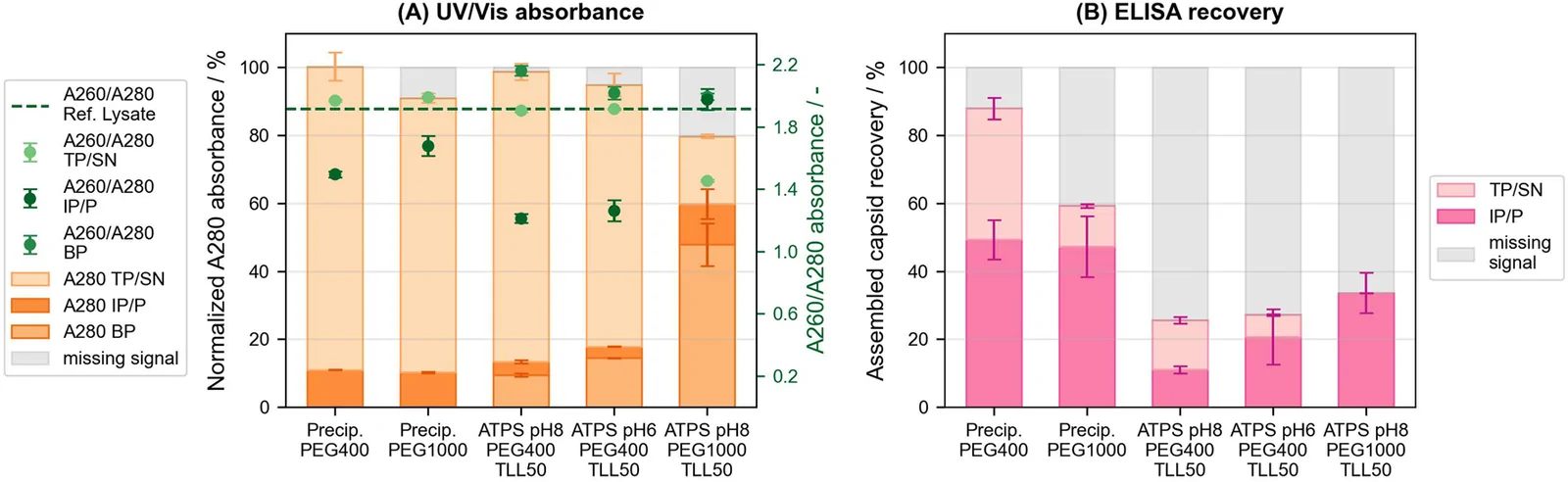
A280-based apparent balance and assembled capsid recovery following resolubilization of selected precipitation and ATPS conditions. **(A)** Normalized UV absorbance at 280 nm is shown for the recovered liquid fractions (top phase, bottom phase, or supernatant; TP/BP/SN) and for the resolubilized solid fractions (interphase or pellet; IP/P). Corresponding A260/A280 absorbance ratios are shown for each recovered fraction, with the clarified lysate indicated as reference. The stacked A280 contributions illustrate the apparent mass balance of soluble biomolecular material after separation and resolubilization. **(B)** Anti-AAV5 ELISA capsid recovery in the corresponding liquid and resolubilized solid fractions. Bottom-phase samples were below the ELISA detection limit and are not included.

For precipitation, the PEG400 condition showed complete A280-based resolubilization of the apparent pellet-associated biomolecular fraction, whereas approximately 53% of this fraction was recovered for PEG1000 (Figure 5A). Capsid-specific ELISA showed a comparable trend (Figure 5B). For PEG400, 39% of the initial capsid signal remained in the supernatant and an additional 49% was recovered from the resolubilized pellet. Relative to the capsid fraction depleted from the supernatant, this corresponds to an apparent capsid resolubilization of approximately 80%. For PEG1000, 12% of the capsids remained in the supernatant and 47% were recovered from the pellet, corresponding to approximately 53% of the apparent pellet-associated capsid fraction. Thus, the capsid-specific results followed the A280-based resolubilization trend, particularly for PEG1000, while PEG400 showed nearly complete biomolecular resolubilization together with high, although not complete, recovery of assembled capsids. Thus, both readouts indicated less favourable overall recovery for PEG1000.

For ATPS, PEG400 systems at pH 8 and pH 6 showed different A280-based apparent balance closure, whereas total ELISA-detectable capsid recovery was similar between the two conditions. At pH 8, a larger capsid fraction remained in the top phase, while at pH 6 a larger fraction was recovered from the resolubilized interphase. PEG1000 at pH 8 showed a larger interphase contribution for both A280 and ELISA, although a substantial fraction of the initial capsid signal remained unaccounted for. Across the three ATPS conditions, approximately 70% of the initial ELISA signal was not recovered. Overall, the amount of ELISA-detectable capsid signal remaining unaccounted for varied substantially among the investigated conditions.

## 4 Discussion

### 4.1 Interpretation and validation of UV-derived particulate-associated scattering

Rapid and high-throughput evaluation of separation conditions is essential for efficiently exploring complex process design spaces and identifying optimal operating windows. The analytical workflow developed in this work enabled screening of 170 precipitation and ATPS system points, performed in biological duplicates, with complete sample preparation, UV/Vis analysis, and data processing completed within 3 days. An automated liquid handling platform enabled rapid preparation and sampling of all conditions, while a SpecPlate allowed simultaneous duplicate UV/Vis measurements at multiple path lengths, minimizing dilution requirements (Radtke, 2026). Automated liquid handling was used in this study to increase experimental throughput and enable systematic exploration of the large design space, but it is not a prerequisite for implementation of the UV-derived readout. The same analytical workflow can be applied to smaller, manually prepared microplate screenings.

The measured UV signal was considered as the sum of a chromophoric absorption term and a scattering contribution arising from suspended particles and aggregates, leading to deviations from the Beer–Lambert law. For clarified lysates, which cannot be described by a single absorptivity due to their compositional complexity, scattering-corrected absorbance values (A260 and A280) were therefore used to estimate the soluble biomolecular fraction. In contrast, wavelengths above 300 nm were exploited to monitor the scattering contribution, where molecular absorbance becomes negligible, and the signal is dominated by suspended particulate species. Consequently, decreases in A320 were interpreted as a reduction in the scattering-active material resulting from precipitation or phase partitioning, whereas persistent or increasing signals were consistent with the persistence or formation of larger scattering-active structures. The capsid titer measured in the clarified lysate confirms the successful expression and assembly of VP3-only AAV-VLPs in *E. coli*, in agreement with previous reports (Le et al., 2019; 2022; Fu et al., 2024). The strong correlation observed between ELISA and A320 during PEG4000 precipitation (Pearson r=0.995 , $R^{2}=0.990$ ) demonstrates that, under the investigated conditions, the UV-derived scattering signal closely tracks capsid depletion from the liquid phase. This relationship was subsequently extended to PEG400 and PEG1000, showing close correspondence at higher PEG molecular weights and increasing divergence toward PEG400.

Interestingly, purified capsids added to clarified non-induced lysate did not produce measurable changes in either A280 or A320 over the investigated concentration range. This observation indicates that dispersed intact capsids alone are not the dominant contributors to the overall biomolecular fraction and UV-derived scattering signal under these conditions. Together with the preliminary precipitation experiments, these findings suggest that the A280 mainly describes host-cell proteins and nucleic acids, while the measured A320 signal predominantly originates from larger scattering-active structures formed during PEG-induced precipitation rather than from individual VLPs. Such structures likely arise from aggregation between VLPs and/or host-cell-derived particulate components, explaining both the transient increase in A320 observed at low precipitant concentrations and its subsequent depletion as these structures partition into the solid phase. This interpretation is also consistent with the constant A280 and higher A320 observed in induced compared with non-induced lysates, suggesting that VLP expression promotes changes in the particulate population, potentially including VLP-containing assemblies. The comparison between induced and non-induced lysates further shows that host-cell-derived particulate material has a measurable contribution to the scattering signal also without VLPs. Consequently, the non-induced control should be regarded as a qualitative reference for background particulate-associated scattering rather than an exact representation of the induced lysate in the absence of VLPs. Differences in host-cell physiology following IPTG induction and potential co-aggregation phenomena may influence the optical response and should therefore be considered when interpreting control-derived corrections.

These results and considerations indicate that this approach does not allow discrimination between individual VLPs, soluble aggregates, or other particulate species present in the lysate, and it should be regarded as a relative indicator of particulate behaviour rather than a direct quantification of VLP concentration. Likewise, the non-linear dependence of light scattering on particle size and concentration precludes direct conversion of A320 into particle mass or quantitative recovery. Accordingly, a capsid-specific detection limit cannot be assigned to A320 from the present data, as its analytical sensitivity refers to changes in the combined particulate-associated scattering signal rather than to capsid titer.

### 4.2 Precipitation screenings

The precipitation performance was evaluated in terms of the differential removal of the host-cell-derived biomolecular fraction (A280) and the particulate-associated scattering signal (A320) as a function of PEG molecular weight (PEG400–PEG1000) under fixed pH (8) and additive concentration (2% NaCl). The observed differences between A280 and A320 profiles indicate that the scattering-active material is depleted from the liquid phase at lower precipitant concentrations than the soluble biomolecular fraction. This behaviour is consistent with the size-dependent nature of PEG-induced precipitation, where larger particulate assemblies experience stronger excluded-volume effects compared to soluble proteins (Asenjo and Andrews, 2012; Tsoka et al., 2000; Pons Royo and Jungbauer, 2025).

The comparison between precipitant agents further highlights the central role of PEG molecular weight on selectivity. Low-molecular-weight PEG400 and PEG600, as well as phosphate-induced precipitation, enabled a more selective removal of scattering-active structures relative to the soluble biomolecular fraction, whereas higher PEG molecular weights led to a more simultaneous removal of both fractions. This observation contrasts with the widespread use of high-molecular-weight PEG (e.g., PEG4000–8000) for viral particle capture, which is known to promote co-precipitation of host-cell impurities (Tsoka et al., 2000; Fu et al., 2024; Pons Royo and Jungbauer, 2025; Fu et al., 2025). While high-molecular-weight PEG systems are effective for bulk recovery at low precipitant concentrations, their limited selectivity has been reported as a bottleneck in downstream processing (Ladd Effio et al., 2015; Pons Royo and Jungbauer, 2025). The present results suggest that lower PEG molecular weights may provide a more pronounced size-selective precipitation mechanism, enabling improved separation between particulate material and soluble contaminants. In phosphate-induced precipitation, Dip’n’Check analysis did not detect capsids despite a pronounced A320 trend. Two non-exclusive explanations need to be considered. First, the high-phosphate matrix may interfere with the immunoassay and thereby underestimate capsid recovery, as high ionic strength and complex buffer compositions can affect immunoassay performance (Zeltins, 2013; Wild, 2013). Alternatively, detectable capsids may already have been depleted at the investigated phosphate concentrations, while the remaining A320 trend reflects continued aggregation and removal of host-cell-derived or other non-VLP particulate material. The present data do not allow these possibilities to be distinguished. Consequently, the phosphate-derived A320 profile should be interpreted exclusively as particulate-associated scattering and not as evidence of continued capsid precipitation.

The concentration-dependent divergence observed for PEG400 provides additional insight into this relationship. At low PEG concentrations, A320 remained elevated or transiently increased despite a decrease in ELISA-detectable capsids. Because scattering is strongly influenced by particle size, this behaviour is consistent with the formation or growth of larger scattering-active assemblies, whose increased optical contribution can compensate for the simultaneous removal of a fraction of the capsids. Conversely, at higher PEG400 concentrations, A320 was strongly depleted while ELISA still detected a substantial capsid fraction in the supernatant. This indicates that removal of the dominant scattering-active structures does not necessarily require complete capsid precipitation under milder, low-molecular-weight PEG conditions. The closer correspondence observed for PEG1000 and particularly PEG4000 therefore reflects a stronger coupling between removal of scattering-active material and capsid depletion under these precipitation regimes. These observations emphasize that UV-derived scattering should be interpreted as a descriptor of particulate-associated scattering rather than capsid concentration itself, while also highlighting its value as a rapid first-line screening tool to identify promising process conditions prior to orthogonal capsid-specific analysis. Future work should therefore investigate whether combining low-molecular-weight PEG with suitable additives or optimized process conditions can increase capsid recovery while preserving its favourable selectivity.

### 4.3 ATPS screenings

Analogous to precipitation screening, UV signals at 280 nm and 320 nm were used to evaluate the behaviour of soluble biomolecular and scattering-active material across the liquid phases (top and bottom) as a function of PEG molecular weight (PEG400–PEG1000), pH (6 and 8), and NaCl concentration (0%–2%). The observed redistribution of A280 and A320 signals across phases highlights the strong dependence of partitioning behaviour on both PEG molecular weight and concentration (tie-line length). In PEG/phosphate systems, these parameters directly influence phase composition and the strength of excluded-volume and salting-out interactions, which govern the distribution of biomolecules and particulate material between phases (Albertsson et al., 1990; Asenjo and Andrews, 2012). In this context, lower molecular weight PEG systems showed a more pronounced separation between soluble biomolecular material and the scattering-active particulate fraction, whereas higher PEG molecular weights promoted a shift of both fractions toward the bottom phase, reducing selectivity, consistent with the work of Fu et al. (2023) and Fu et al. (2025). This behaviour can be attributed to the increased hydrophobicity and excluded-volume effects of larger PEG chains, which enhance phase incompatibility and modify the partitioning environment (Huddleston et al., 1996; Fu et al., 2025). The increase in interphase-associated material upon NaCl addition suggests enhanced non-specific aggregation or precipitation, consistent with the strengthening of salting-out effects at higher ionic strength (Von Hippel and Schleich, 1969). Similarly, the changes observed between pH 8 and 6 indicate altered stability of particulate species. This behaviour is in agreement with previously reported stability profiles of VP3-only AAV systems, which show reduced capsid stability near their isoelectric point of 6.3 (Venkatakrishnan et al., 2013). Under these conditions, reduced colloidal stability may promote the formation of larger scattering-active structures that accumulate in the interphase rather than their partitioning into the liquid phases. Capsid destabilization may contribute to this behaviour, but A320 alone cannot establish the capsid-specific contribution.

ELISA analysis of selected ATPS samples showed a progressive decrease in detectable capsid recovery in the top phase with increasing PEG molecular weight. This trend supports a PEG-molecular-weight-dependent redistribution of capsids away from the top phase, consistent with the increasing strength of the partitioning and precipitation effects observed by UV analysis. All analysed bottom-phase samples were below the ELISA detection limit. However, because similar negative results were obtained in phosphate-rich precipitation samples despite clear UV-derived scattering trends, matrix effects associated with high salt concentrations may contribute to an underestimation of capsid recovery in the bottom phase, but a true low abundance of intact capsids in these fractions cannot be excluded.

The lower A260/A280 ratio in the top phase, together with the comparatively constant ratio in the bottom phase, is consistent with a greater relative nucleic-acid-associated contribution in the bottom phase. This behaviour is consistent with previous observations in PEG/salt systems, where nucleic acids exhibit higher affinity for the salt-rich phase due to their charge density and interactions with the ionic environment (Albertsson et al., 1990; Johansson et al., 2012). Overall, these results suggest that precipitation phenomena play a central role in governing phase partitioning in PEG/phosphate ATPS systems, leading to the accumulation of scattering-active structures in the interphase while soluble biomolecular material preferentially partitions into the coexisting liquid phases.

### 4.4 Process reversibility and resolubilization

Finally, resolubilization experiments were performed to evaluate the reversibility of the separation processes. In this context, the UV signal at 280 nm was used as a relative indicator of the soluble biomolecular fraction recoverable after resolubilization. As A280 cannot distinguish intact capsids, dissociated VP3 and other soluble components, capsid-specific ELISA measurements were introduced. These measurements provide a capsid-specific, conformation-dependent assessment of the resolubilized material. Since the capture antibody recognizes a conformational epitope present on assembled AAV5 capsids but absent from unassembled capsid proteins, recovery of ELISA signal confirms that at least a fraction of the precipitated or interphase-associated capsids remained or returned to an assembled state following resolubilization. However, the assay does not provide a complete structural characterization of the recovered particles, and loss of ELISA signal cannot distinguish between irreversible capsid loss, aggregation, conformational changes affecting epitope recognition, or matrix-dependent assay interference. Accordingly, these experiments should be interpreted as a feasibility assessment of process reversibility rather than as a comprehensive characterization of capsid structure and functionality.

For PEG400 precipitation, although the A280-based apparent balance was essentially complete after resolubilization, only a fraction of the initial capsid signal was recovered from the pellet by ELISA. PEG1000 showed both incomplete A280-based balance closure and, in a similar ratio, a larger overall deficit in ELISA-detectable capsid recovery, suggesting a less favorable overall recovery under the tested conditions. The lower recovery observed for PEG1000 is qualitatively consistent with the stronger excluded-volume effects associated with higher PEG molecular weight. Similar trends have been reported in polymer-induced precipitation systems, where higher PEG molecular weights promote tighter association of biomolecules and reduced resolubilization efficiency (Asenjo and Andrews, 2012; Ladd Effio et al., 2015).

For ATPS, A280-based interpretation suggests lower reversibility at pH 6 than at pH 8. Capsid-specific analysis shows that PEG400 systems at pH 6 and pH 8 have similar total ELISA-detectable capsid recovery but a markedly different distribution between the liquid phase and resolubilized interphase, with pH 6 shifting a larger fraction of detectable capsids toward the interphase. Thus, the lower liquid-phase scattering signal observed at pH 6 should not be interpreted simply as reduced capsid stability. Rather, pH affects both particulate-associated behaviour and the phase distribution of capsids. PEG1000 ATPS showed a larger fraction of both A280 and ELISA-detectable capsids associated with the resolubilized interphase, but substantial A280 signal remained unaccounted for, showing reduced solubility of the pellet fraction. Across all ATPS conditions evaluated by ELISA, approximately 70% of the initial capsid signal remained missing.

Overall, these experiments demonstrate that capsid-specific analysis remains necessary after UV-based identification of promising process regions. The aim of the present study was not to establish an optimized capsid resolubilization protocol or to comprehensively characterize post-resolubilization capsid stability, but to evaluate UV-derived scattering as a high-throughput first-line screening tool capable of reducing the number of samples requiring such detailed orthogonal characterization. The operating windows identified in this study are specific to the investigated VP3-only AAV5/*E. coli* lysate system and should not be interpreted as universal conditions for other AAV serotypes, VLPs, or process matrices. Rather, the value of the proposed workflow lies in facilitating rapid, system-specific process optimization while restricting capsid-specific characterization to a smaller set of selected conditions. Future work will investigate the structural state of recovered capsids in greater detail and determine the contributions of aggregation, urea exposure, and high-salt/phosphate matrix effects to capsid recovery measurements, including evaluation of alternative ligands and orthogonal quantification methods.

## 5 Conclusion and outlook

In this study, UV-derived scattering was established as a rapid comparative readout for high-throughput screening of VLP separation conditions in complex process samples. A320 did not directly report capsid concentration, but reflected larger scattering-active structures whose depletion showed condition-dependent agreement with capsid-specific ELISA. This agreement was strongest for higher-molecular-weight PEG and became increasingly decoupled at lower PEG molecular weight, while capsid-spiking experiments confirmed that dispersed AAV5 capsids alone contribute only marginally to the signal. The workflow enabled evaluation of 170 precipitation and PEG/phosphate ATPS conditions in biological duplicate within 3 days. Across this design space, PEG molecular weight strongly influenced separation behaviour, with low-molecular-weight PEG providing greater differentiation between particulate-associated scattering and the soluble biomolecular fraction, although capsid-specific analysis showed that further optimization is required to improve assembled-capsid recovery. The identified operating windows are specific to the investigated product and matrix, while automation increased screening throughput but is not required for implementation of the analytical readout.

The resolubilization experiments further supported a sequential analytical strategy in which UV-derived signals are used to identify process regions of interest and capsid-specific methods are subsequently applied to selected conditions. A280 provided a rapid assessment of soluble biomolecular recovery, while conformation-dependent ELISA enabled evaluation of assembled-capsid recovery after resolubilization. These experiments should therefore be considered a feasibility assessment of process reversibility rather than a complete characterization of capsid stability. Overall, the proposed workflow can reduce the analytical burden associated with large process design spaces by restricting detailed capsid-specific and structural characterization to a smaller subset of samples. Future work will focus on improving capsid recovery under selective low-molecular-weight PEG conditions and on distinguishing true capsid losses from structural changes and matrix effects associated with urea- and phosphate-rich samples.

## Data Availability

The original contributions presented in the study are included in the article/Supplementary Material, further inquiries can be directed to the corresponding author.
