# Supplementary material for: UV-derived scattering as a high-throughput readout for adeno-associated virus-like particle separation screenings
Source: Front Bioeng Biotechnol. 2026 Oct 13;14:1913731. doi: 10.3389/fbioe.2026.1913731 (PMC13710323; doi:10.3389/fbioe.2026.1913731)
Supplement: Supplementary file 1 [file DataSheet1.pdf]

Supplementary Material

1 VIRUS-LIKE PARTICLE PRODUCTION IN *ESCHERICHIA COLI*

A 1 L LB medium containing 50 µg/mL kanamycin in a 2 L shake flask was inoculated to an initial OD_{600} of 0.1 (Implen OD600®, Implen GmbH, München, Germany) and incubated at 37°C and 170 rpm for approximately 2.5 h, until an optical density OD_{600} of 0.9 - 1.2 was reached. VP3 expression was induced by the addition of IPTG to a final concentration of 0.4 mM, and the temperature was reduced to 20°C. After overnight incubation at 170 rpm, a final OD_{600} of approximately 4 was reached. Cells were harvested by centrifugation at 4 °C and 3220 $\times g$ for 20 min using an Eppendorf Centrifuge 5810 R (Eppendorf SE, Hamburg, Germany), and pellets were washed with lysis buffer. For lysis, pellets from 1 L culture volume were thawed and resuspended in lysis buffer (30 mL for an OD_{600} of 4) in 50 mL centrifugal tubes (Protein LoBind®, Eppendorf SE, Hamburg, Germany). Cell disruption was performed by three rounds of ultrasonic treatment using a Digital Sonifier 550 (Branson Ultrasonic Corporation, Brookfield, US-CT) in a stirred ice bath (70% amplitude, 10 s pulse, 30 s pause, 20 cycles per round).

The composition of the clarified lysate was assessed using SDS-PAGE (Figure S1). In the region between 55.4 kDa and 66.3 kDa, multiple bands are observed, making it difficult to assign a specific band to the expressed VP3 protein (59.6 kDa). The influence of PEG molecular weight in the protein distribution is shown in lanes 3-4 (PEG400) and lanes 7-8 (PEG1000), where a shift of proteins from the top phase to the bottom phase can be seen.

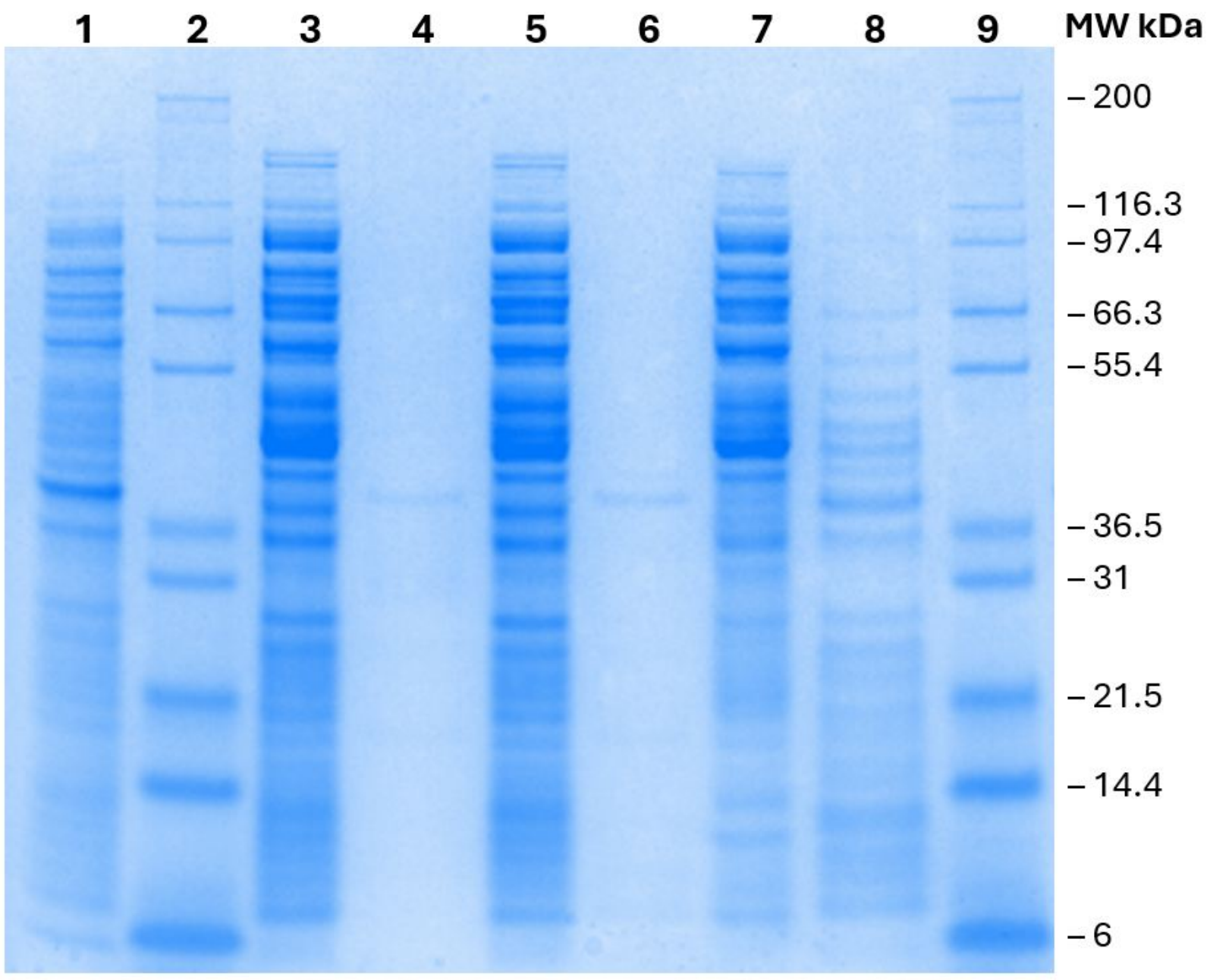

Figure S1. Coomassie-stained SDS-PAGE of clarified lysate and selected ATPS process samples. Protein composition of the clarified lysate (lane 1), protein standard (lanes 2 and 9), PEG400 TLL50 pH8 top (lane 3) and bottom (lane 4) phases, PEG400 TLL50 pH6 top (lane 5) and bottom (lane 6) phases, PEG1000 TLL50 pH8 top (lane 7) and bottom (lane 8) phases. Dilution of all the samples was kept constant.

2 ATPS BINODALS AND TIE-LINES DETERMINATION

To extend the analysis to two-phase systems, ATPS characterization was performed to define suitable phase compositions for partitioning experiments. Phase diagrams for sixteen different PEG/salt systems were generated using automated routines on a liquid handling station (see Figure S2 and Table S1). The resulting binodal curves are consistent with previously reported data (Ladd Effio and Hubbuch, 2015; Meutelet et al., 2024), confirming the reliability of the automated approach developed by Oelmeier et al. (Oelmeier et al. (2011)). An increase in PEG molecular weight resulted in a shift of the binodal curves toward lower component concentrations, while the addition of NaCl or adjustment of the pH to 6 further shifted the binodal curves, in agreement with literature reports (Glyk et al., 2014; Asenjo and Andrews, 2012).

Tie-lines were determined using a colorimetric method with water, phosphate, PEG, additive, and methyl orange as the colorant, as developed by Diederich et al. (2013) and adapted by Meutelet et al. (2024), with modifications. First, points in the center of the two-phase region of the binodal plot were randomly selected to obtain tie-lines evenly distributed across the region. The average slope was then used to calculate theoretical tie-lines with predefined lengths ranging from 20 to 55, in steps of 5. Finally, the calculated system compositions of the theoretical tie-lines with V_R equal to 1 were used as input for the automated tie-line determination method described above, yielding the final system compositions and tie-lines used in this study. For each ATPS systems, eight system points corresponding to tieline lengths (TLLs) ranging from 20 to 55 were created. All the calculated points can be found in Tables S2 to S5.

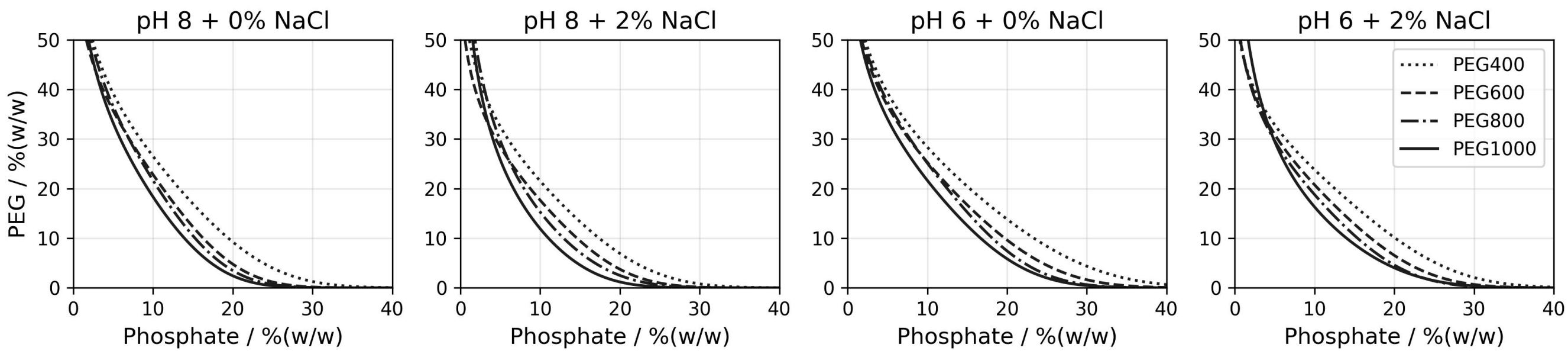

Figure S2. Binodal curves for PEG/phosphate ATPS at different pH, with 0-2% NaCl as an additive and with various PEG molecular weights. Curves were obtained using an automated tritrimetric method, and data were fitted using Equation 1.

Table S1. Binodal coefficients a, b, and c of the investigated PEG/phosphate ATPS varying on polymer molecular weight (MW), pH, and additive (NaCl).

PEG MW	pH	Additive %(w/w)	A	B	C
400	8	0	84.81700	-0.34045	-0.00009
600	8	0	76.21141	-0.33026	-0.00016
800	8	0	89.28557	-0.38856	-0.00019
1000	8	0	93.07079	-0.44888	-0.00021
400	8	2	72.75938	-0.35501	-0.00010
600	8	2	66.16865	-0.36963	-0.00015
800	8	2	108.17717	-0.57060	-0.00015
1000	8	2	109.29031	-0.63406	-0.00021
400	6	0	79.31455	-0.31112	-0.00004
600	6	0	77.49332	-0.32973	-0.00008
800	6	0	78.84277	-0.32567	-0.00011
1000	6	0	80.87923	-0.38015	-0.00012
400	6	2	62.48054	-0.28328	-0.00007
600	6	2	65.99858	-0.33294	-0.00010
800	6	2	68.52271	-0.36694	-0.00013
1000	6	2	101.91557	-0.54847	-0.00010

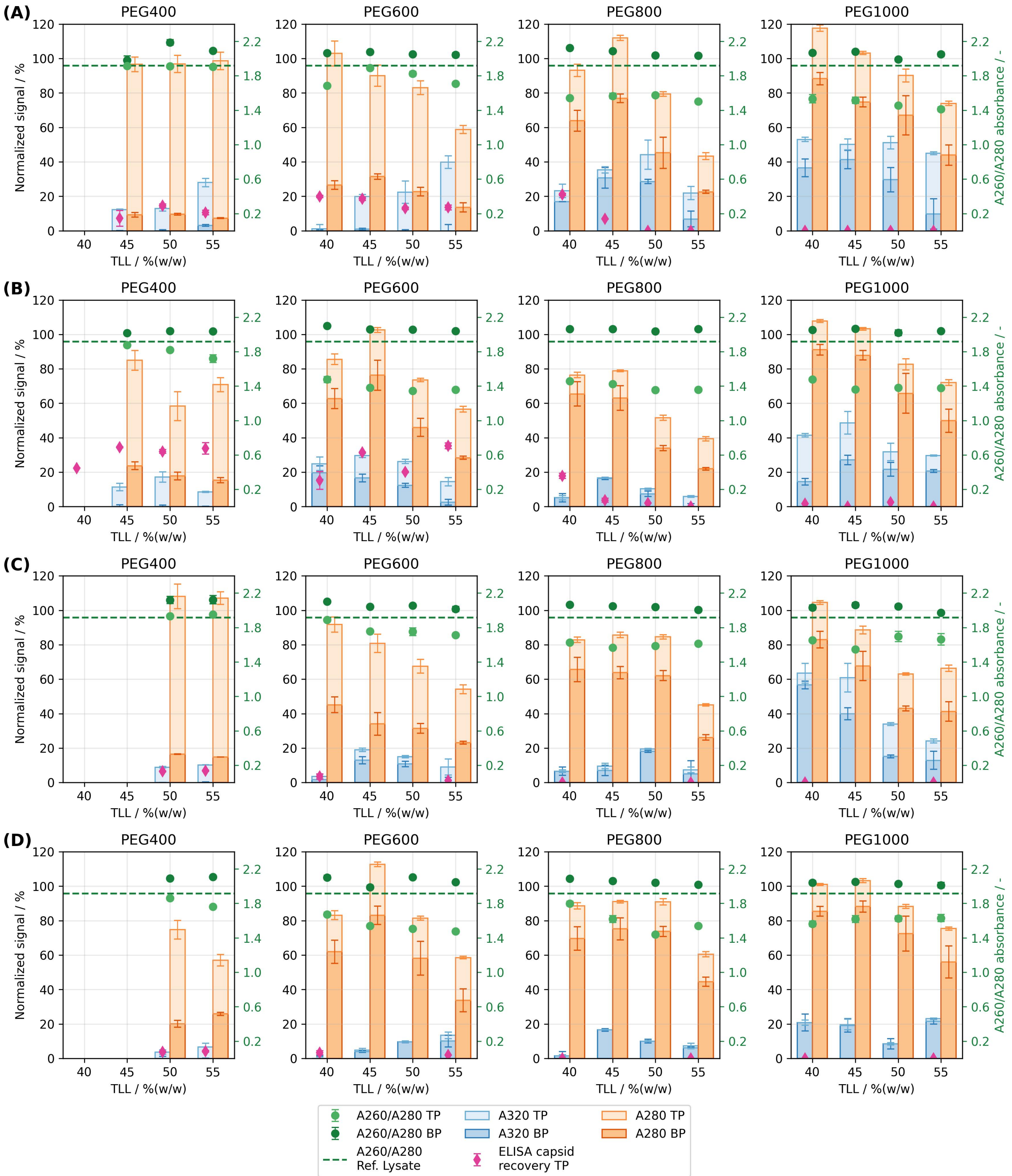

Figure S3. Partitioning of UV-derived signals in top phase (TP) and bottom phase (BP) for PEG/phosphate ATPS systems at increasing tie-line length (TLL). **(A)** pH 8, 0% NaCl; **(B)** pH 8, 2% NaCl; **(C)** pH 6, 0% NaCl; **(D)** pH 6, 2% NaCl. Bars represent normalized protein (A280) and particulate (A320) signals in each phase, while the A260/A280 ratio indicates the relative contribution of nucleic acids to total protein content. TLLs where two phases were not achieved are not represented, or represented as empty.

Table S2. System points of the two-phase systems with PEG400, PEG600, PEG800 and PEG1000 at pH 8 without additives. For each system point, the respective polymer, phosphate, and additive concentrations are listed, along with the calculated tie-line lengths (TLL) and slopes (TLS).

System Point	1	2	3	4	5	6	7	8
PEG 400 %(w/w)	13.727	12.518	15.772	16.629	17.436	18.513	20.604	22.252
Phosphate %(w/w)	17.815	19.279	17.615	17.842	18.302	18.803	18.928	20.340
TLL	20.332	23.645	30.342	34.917	39.262	43.741	49.478	55.519
TLS	-1.488	-1.346	-1.485	-1.460	-1.418	-1.381	-1.399	-1.320
PEG 600 %(w/w)	13.720	15.073	16.061	17.023	17.606	18.753	21.453	22.993
Phosphate %(w/w)	15.317	15.111	15.194	15.416	16.047	16.584	17.181	19.493
TLL	23.040	28.334	32.733	36.802	40.117	44.118	51.297	57.389
TLS	-1.751	-1.749	-1.717	-1.675	-1.590	-1.530	-1.507	-1.339
PEG 800 %(w/w)	14.829	15.984	17.025	17.958	18.795	19.710	21.922	23.642
Phosphate %(w/w)	13.634	13.514	13.554	13.776	14.212	14.826	15.760	17.875
TLL	21.181	26.347	31.149	35.576	39.621	43.502	50.126	56.224
TLS	-2.037	-2.041	-2.024	-1.986	-1.919	-1.835	-1.749	-1.552
PEG 1000 %(w/w)	13.664	14.811	15.967	17.277	18.595	19.485	22.545	23.594
Phosphate %(w/w)	12.921	12.864	12.948	13.120	13.475	14.281	14.667	16.150
TLL	20.105	25.215	30.159	35.055	39.709	43.525	50.816	54.689
TLS	-1.950	-1.960	-1.953	-1.943	-1.913	-1.812	-1.855	-1.699

Table S3. System points of the two-phase systems with PEG400, PEG600, PEG800, and PEG1000 at pH 8 with 2 %(w/w) NaCl as an Additive. For each system point, the respective polymer, phosphate, and additive concentrations are listed, along with the calculated tie-line lengths (TLL) and slopes (TLS).

System Point	1	2	3	4	5	6	7	8
PEG 400 %(w/w)	12.941	14.997	15.913	16.168	16.104	19.196	19.919	21.305
Phosphate %(w/w)	12.941	15.266	15.342	16.091	17.438	16.499	17.557	19.143
TLL	19.823	25.754	30.709	34.939	38.698	44.974	48.757	54.186
TLS	-1.367	-1.437	-1.436	-1.377	-1.266	-1.402	-1.336	-1.258
PEG 600 %(w/w)	13.821	15.029	15.784	16.445	17.694	19.233	20.752	22.692
Phosphate %(w/w)	13.043	12.833	13.080	13.628	14.203	15.441	16.312	17.977
TLL	20.911	26.172	30.870	35.321	40.393	46.304	50.729	56.612
TLS	-1.579	-1.601	-1.584	-1.540	-1.510	-1.433	-1.405	-1.339
PEG 800 %(w/w)	13.990	15.401	16.932	17.356	18.876	20.240	20.654	22.082
Phosphate %(w/w)	11.779	11.703	11.765	12.585	12.679	13.921	17.662	19.080
TLL	19.838	25.130	30.555	34.917	39.036	44.895	50.821	55.279
TLS	-1.819	-1.887	-1.948	-1.855	-1.913	-1.803	-1.391	-1.328
PEG 1000 %(w/w)	13.214	13.787	15.367	17.584	17.670	20.004	22.366	23.958
Phosphate %(w/w)	11.054	11.122	11.285	11.364	12.727	13.068	14.790	16.025
TLL	22.595	24.844	30.128	35.998	39.199	44.673	51.470	55.826
TLS	-1.854	-1.870	-1.920	-2.024	-1.802	-1.870	-1.743	-1.670

Table S4. System points of the two-phase systems with PEG400, PEG600, PEG800 and PEG1000 at pH 6 without additives. For each system point, the respective polymer, phosphate, and additive concentrations are listed, along with the calculated tie-line lengths (TLL) and slopes (TLS).

System Point	1	2	3	4	5	6	7	8
PEG 400 %(w/w)	10.498	13.171	13.835	14.268	14.963	15.676	16.597	17.344
Phosphate %(w/w)	24.163	22.218	22.396	22.990	23.579	24.449	25.427	27.044
TLL	19.346	25.443	30.153	34.525	39.052	43.560	48.277	53.105
TLS	-0.988	-1.084	-1.069	-1.033	-1.000	-0.957	-0.917	-0.855
PEG 600 %(w/w)	12.365	13.550	14.503	15.702	16.391	16.986	18.225	19.399
Phosphate %(w/w)	19.164	18.930	19.011	19.075	19.743	20.801	21.606	22.793
TLL	21.640	26.912	31.632	36.442	40.459	44.319	48.895	53.488
TLS	-1.269	-1.275	-1.261	-1.254	-1.205	-1.135	-1.098	-1.049
PEG 800 %(w/w)	13.157	14.550	15.949	16.558	17.470	17.927	18.970	19.795
Phosphate %(w/w)	17.360	17.093	16.927	17.374	17.821	18.856	19.778	21.262
TLL	22.522	27.966	33.094	36.820	40.783	44.023	48.056	52.031
TLS	-1.533	-1.539	-1.539	-1.484	-1.436	-1.337	-1.268	-1.170
PEG 1000 %(w/w)	11.880	13.291	14.359	15.358	17.576	18.775	19.856	21.043
Phosphate %(w/w)	16.500	16.161	16.236	16.563	16.264	16.922	17.948	19.192
TLL	19.662	24.939	29.805	34.408	40.176	44.647	48.826	53.105
TLS	-1.425	-1.453	-1.442	-1.413	-1.470	-1.429	-1.362	-1.292

Table S5. System points of the two-phase systems with PEG400, PEG600, PEG800, and PEG1000 at pH 6 with 2 %(w/w) NaCl as an Additive. For each system point, the respective polymer, phosphate, and additive concentrations are listed, along with the calculated tie-line lengths (TLL) and slopes (TLS).

System Point	1	2	3	4	5	6	7	8
PEG 400 %(w/w)	9.359	12.245	14.703	14.733	15.464	15.961	16.368	16.654
Phosphate %(w/w)	22.242	20.318	19.057	19.946	20.488	21.710	24.262	25.857
TLL	19.926	27.297	34.125	37.147	41.082	45.043	50.389	53.661
TLS	-0.984	-1.076	-1.131	-1.073	-1.039	-0.972	-0.851	-0.791
PEG 600 %(w/w)	10.373	13.453	13.768	14.723	16.205	17.258	18.623	18.864
Phosphate %(w/w)	18.329	16.513	16.833	17.086	17.656	18.518	19.449	21.899
TLL	21.127	27.758	30.495	34.860	40.731	45.028	49.653	53.679
TLS	-1.159	-1.288	-1.258	-1.237	-1.204	-1.157	-1.124	-0.987
PEG 800 %(w/w)	12.497	13.749	14.827	15.711	16.632	17.853	19.690	21.202
Phosphate %(w/w)	14.955	14.786	14.911	15.391	16.138	16.994	17.653	18.839
TLL	22.130	27.316	32.039	36.322	40.482	44.876	49.852	54.491
TLS	-1.415	-1.425	-1.416	-1.379	-1.325	-1.278	-1.276	-1.237
PEG 1000 %(w/w)	12.299	13.665	14.849	16.158	17.942	19.512	20.350	21.709
Phosphate %(w/w)	13.929	13.905	14.259	14.773	15.224	16.041	17.552	18.927
TLL	20.063	25.383	30.545	35.636	40.988	45.963	49.978	54.463
TLS	-1.384	-1.435	-1.449	-1.453	-1.484	-1.472	-1.369	-1.312

REFERENCES

Asenjo, J. A. and Andrews, B. A. (2012). Aqueous two-phase systems for protein separation: Phase separation and applications. *Journal of Chromatography A* 1238, 1–10. doi:10.1016/j.chroma.2012.03.049

Diederich, P., Amrhein, S., Hämmerling, F., and Hubbuch, J. (2013). Evaluation of PEG/phosphate aqueous two-phase systems for the purification of the chicken egg white protein avidin by using high-throughput techniques. *Chemical Engineering Science* 104, 945–956. doi:10.1016/j.ces.2013.10.008

Glyk, A., Scheper, T., and Beutel, S. (2014). Influence of Different Phase-Forming Parameters on the Phase Diagram of Several PEG–Salt Aqueous Two-Phase Systems. *Journal of Chemical & Engineering Data* 59, 850–859. doi:10.1021/je401002w

Ladd Effio, C. and Hubbuch, J. (2015). Next generation vaccines and vectors: Designing downstream processes for recombinant protein-based virus-like particles. *Biotechnology Journal* 10, 715–727. doi:10.1002/biot.201400392

Meutelet, R., Bisch, L. J., Buerfent, B. C., Müller, M., and Hubbuch, J. (2024). Partitioning behavior of short DNA fragments in polymer/salt aqueous two-phase systems. *Biotechnology Journal* 19, 2400394. doi:10.1002/biot.202400394

Oelmeier, S. A., Dismer, F., and Hubbuch, J. (2011). Application of an aqueous two-phase systems high-throughput screening method to evaluate mAb HCP separation. *Biotechnology and Bioengineering* 108, 69–81. doi:10.1002/bit.22900
